# K63-linked ubiquitylation induces global sequestration of mitochondria

**DOI:** 10.1038/s41598-020-78845-7

**Published:** 2020-12-18

**Authors:** Thibaud J. C. Richard, Laura K. Herzog, Julia Vornberger, Aldwin Suryo Rahmanto, Olle Sangfelt, Florian A. Salomons, Nico P. Dantuma

**Affiliations:** Department of Cell and Molecular Biology, Biomedicum, Karolinska Institutet, Solnavägen 9, 17177 Stockholm, Sweden

**Keywords:** Biological techniques, Cell biology

## Abstract

Even though K63-linked polyubiquitin chains do not target proteins for proteasomal degradation, they play nevertheless a complementary protective role in maintaining protein homeostasis by directing malfunctioning proteins and organelles to inclusion bodies or autophagosomes. A paradigm for this process is the sequestration and autophagic degradation of dysfunctional mitochondria. Although studies have shown that K63-ubiquitylation of mitochondrial proteins by the ubiquitin ligase Parkin is important in this process, it is presently not clear if this modification also suffices to initiate this cascade of events. To address this question, we have engineered the ubiquitin ligase ProxE3, which in an inducible manner synthesizes K63-linked ubiquitin chains on the surface of mitochondria. We found that the presence of K63-linked ubiquitin chains on mitochondria resulted in the recruitment of the ubiquitin adaptor p62 and induced a dramatic redistribution of mitochondria, which was reminiscent to the Parkin-facilitated sequestration in response to mitochondrial uncoupler. However, ProxE3 did not induce autophagic degradation of mitochondria. Our data show that K63-linked ubiquitin chains at the mitochondrial membrane are sufficient for the induction of mitochondrial sequestration, but not mitophagy, without the need of extrinsically inflicting mitochondrial dysfunction.

## Introduction

The identification of new functions of ubiquitin modifications has been expanding during recent years, implicating this protein modifier, which is best known for its role in targeting proteins for proteasomal degradation^1^, in a plethora of cellular processes. This has also increased the awareness that polyubiquitylation cannot be looked upon as a single posttranslational modification as different ubiquitin chain compositions, which are a direct consequence of the various lysine residues in ubiquitin used to build chains, result in a complex ubiquitin code that is presently only poorly understood^2^. Among the different ubiquitin modifications, K63-linked ubiquitin chains are exceptional as they are the only ubiquitin chains that do not target proteins for proteasomal degradation in the natural intracellular environment^3,4^. Instead, this type of chains has been linked, among other functions, to the formation of inclusion bodies^5^ and macroautophagy^6^, although it has been debated if their presence within these subcellular structures is a cause or consequence of the sequestration of misfolded proteins or organelles^7^.

The targeting of dysfunctional mitochondria to perinuclear clusters and their degradation by autophagy, i.e. mitophagy, are intimately linked to K63-linked ubiquitylation of mitochondrial surface proteins. The ring-between-ring (RBR) ubiquitin ligase Parkin translocates to depolarized mitochondria and decorates the dysfunctional mitochondria with K63-linked ubiquitin chains by modifying a number of proteins in the mitochondrial outer membrane (MOM)^8^. This results in the recruitment of the ubiquitin receptor p62 that bridges the mitochondria to the dynein transport machinery facilitating retrograde transport to aggresome-like structures in the perinuclear region^6,9^. Parkin-mediated ubiquitylation also stimulates the sequestration and retrograde transport of misfolded proteins, suggesting a more generic role in targeting of dysfunctional and potentially toxic cellular constituents to inclusion bodies^10^. In a parallel Parkin-dependent pathway, which involves the autophagy receptors OPTN and NDP52^11^ and the autophagy proteins ULK1, FIP200 and ATG9^12–14^, dysfunctional mitochondria are targeted for degradation by mitophagy. Thus, Parkin-mediated ubiquitylation of mitochondrial proteins appears to play a dual role since it regulates the OPTN/NDP52-facilitated targeting of mitochondria for autophagy^11^, independent from its ability to stimulate p62-dependent targeting of mitochondria for retrograde transport^15,16^.

It is, however, presently unclear if K63-linked ubiquitin chains are also sufficient to initiate this process as, in the context of damaged mitochondria, this ubiquitin mark is part of a more complex response involving a number of post-translational modifications. Loss of mitochondrial membrane potential causes stabilization of the serine/threonine kinase PINK1 at the mitochondria^17^, where it phosphorylates the ubiquitin-like domain of Parkin^18–20^, as well as ubiquitin itself^21,22^. Moreover, Parkin not only generates K63-linked chains but also other polyubiquitin chains, including K48-linked ubiquitin chains that target MOM proteins for proteasomal degradation. This process may also promote sequestration and/or autophagy by preventing fusion of dysfunctional mitochondria^23^, and exposing mitochondrial inner membrane (MIM) proteins^24,25^.

Here we studied whether the presence of K63-linked ubiquitin chains suffices to induce sequestration of undamaged mitochondria. To probe into this question, we generated an engineered ubiquitin ligase that, in an inducible manner, translocates to mitochondria where it conjugates specifically K63-linked ubiquitin chains to a reference substrate localized at the MOM. This allowed us to investigate the direct effect of the presence of K63-linked ubiquitin chains at the surface of mitochondria in the absence of other signals triggered by mitochondrial dysfunction. Interestingly, K63-linked ubiquitin chains were sufficient to induce perinuclear sequestration of mitochondria, mimicking the mitochondrial redistribution in Parkin-expressing cells in response to loss of mitochondrial membrane potential, but failed to induce mitophagy. Our data show that K63-linked ubiquitin chains are sufficient for triggering retrograde transport and sequestration of mitochondria and suggest that additional signals are required for induction of mitophagy.

## Results

### ProxE3: an engineered ubiquitin ligase for inducible K63-linked polyubiquitylation

With the purpose of determining the biological effect of K63-linked polyubiquitin chains, we initiated the development of an experimental system that would allow K63-linked ubiquitylation of a reference protein in living cells. To avoid confounding factors that could directly or indirectly change the behavior of the modified proteins, we argued that the system had to fulfill several criteria. Firstly, the target for K63-linked ubiquitylation should be a reference substrate without any specific biological functions. Secondly, the ubiquitin ligase should modify the reference substrate selectively with K63-linked ubiquitin chains. Thirdly, the engineered ubiquitin ligase should lack endogenous substrates to avoid indirect effects caused by K63-linked ubiquitylation of proteins other than the reference substrate. Fourthly, ubiquitylation by the ligase should be inducible allowing regulation of the ubiquitylation status of the reference substrate.

**Figure 1:**
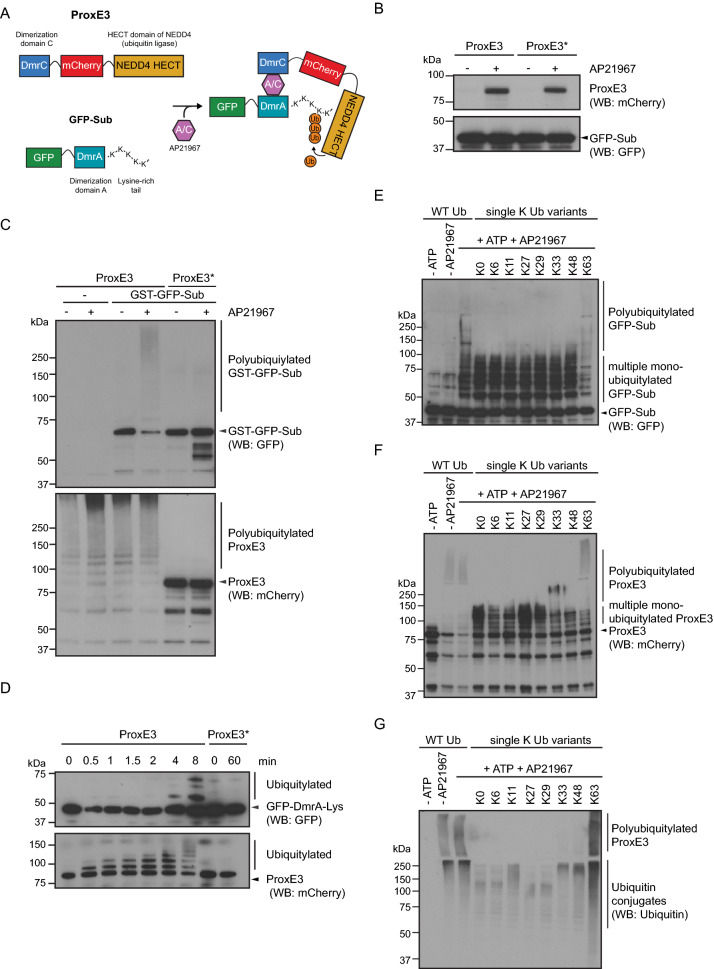
ProxE3: an engineered ubiquitin ligase for inducible K63-linked polyubiquitylation. (**A**) Schematic illustration of the ProxE3 ligase and its substrate GFP-Sub. Dimerization of the DmrC domain in ProxE3 and the DmrA domain in GFP-Sub is induced by the heterodimerizer AP21967 and results in specific K63-linked ubiquitylation of GFP-Sub. (**B**) In vitro heterodimerization of GFP-Sub and mCherry-ProxE3. Recombinant GFP-Sub was immobilized on GFP-Trap magnetic beads prior to addition of mCherry-ProxE3 in the presence or absence of AP21967. (**C**) One-hour in vitro ubiquitylation reaction using mCherry-ProxE3 or the catalytically inactive mCherry-ProxE3* in the presence or absence of AP21967, with or without immobilized GST-tagged GFP-Sub. (**D**) In vitro ubiquitylation kinetics of GFP-Sub by mCherry-ProxE3 or the catalytically inactive mCherry-ProxE3* and auto-ubiquitylation of the ligases. (**E**–**G**) One-hour in vitro ubiquitylation reaction of GFP-Sub by mCherry-ProxE3 with WT ubiquitin (WT Ub), a lysine-less ubiquitin mutant(UbK0) or single lysine ubiquitin mutants. Arrows are pointing towards the unmodified protein of interest (either GFP-Sub or ProxE3). (**E**) GFP detection, Western blot. (**F**) mCherry detection, Western blot. (**G**) Ubiquitin detection, Western blot.

To accomplish this, we based our engineered ubiquitin ligase on the HECT domain of the K63-specific ubiquitin ligase NEDD4. By using the catalytic domain of NEDD4 (^NEDD4^HECT) only, we aimed to avoid interaction of the engineered ligase with endogenous NEDD4 substrates. We opted for a HECT domain as HECT ubiquitin ligases, unlike RING ubiquitin ligase, determine the nature of the ubiquitin chains independent of the ubiquitin conjugase involved in the process^26^. We generated the fusion protein with the wild-type ^NEDD4^HECT domain as well as a catalytically inactive variant, ^NEDD4^HECT^C867S^, in which the catalytic cysteine residue had been substituted for a serine. The active and inactive ubiquitin ligases were expressed with an mCherry tag. As a substrate, we selected the enhanced green fluorescent protein (EGFP)^27^. EGFP was provided with a lysine-rich C-terminal tail to ensure the presence of easily accessible ubiquitylation sites for the engineered HECT ubiquitin ligase. The ubiquitin ligase and substrate were fused to the DmrC and DmrA domains of FKBP12, respectively, which allowed tight regulation of their interaction by administration of the ligand AP21967^28^. We coined the active ubiquitin ligase ProxE3 (Proximity-induced E3 ubiquitin ligase), the inactive ubiquitin ligase ProxE3* and the EGFP-based substrate GFP-Sub (Fig. 1A). Immunoprecipitation experiments confirmed that recombinant ProxE3 and ProxE3* only interacted with GFP-Sub in the presence of the ligand AP21967 (Fig. 1B). After demonstrating direct physical interaction of ProxE3 with GFP-Sub in the presence of AP21967, we analyzed if inducing the interaction resulted in ubiquitylation of GFP-Sub in vitro. The ProxE3 or ProxE3* ligases were first pre-incubated for 30 min with GFP-Sub in the presence or absence of AP21967. Directly after the pre-incubation, the ubiquitylation reaction was started by administration of recombinant HA-tagged ubiquitin (HA-Ub) and ATP. Ubiquitylation of GFP-Sub occurred upon dimerization with ProxE3, as indicated by the high molecular weight products that were detected with a GFP antibody only in the presence of AP21967 (Fig. 1C, upper panel). As expected, ubiquitylation was depending on the catalytic activity of ProxE3 as these modified species were absent when GFP-Sub was incubated with the inactive ligase ProxE3* instead. Notably, we detected also autoubiquitylation of ProxE3, which was independent of the presence of the substrate or administration of AP21967 (Fig. 1C, lower panel). Autoubiquitylation of ProxE3 occurred already within 30 s and was considerably faster than ubiquitylation of GFP-Sub, which became apparent 4–8 min after administration of AP21967 (Fig. 1D).

To determine the nature of the ubiquitin chains generated by ProxE3, we performed the in vitro ubiquitylation reaction in the presence of a panel of HA-tagged ubiquitin variants in which lysine residues had been substituted with arginine residues. While in the presence of wild-type HA-Ub we observed high-molecular weight polyubiquitin chains conjugated to GFP-Sub in an ATP- and AP21967-dependent fashion, HA-tagged ubiquitin lacking lysine residues (HA-Ub^K0^), which does not support the formation of chains, gave rise to smaller products containing up to seven ubiquitin moieties (Fig. 1E). This suggests that GFP-Sub can be multi-monoubiquitylated at least at seven lysine residues.

Performing the in vitro ubiquitylation reaction with seven HA-Ub variants each containing only one of the natural lysine residues present in ubiquitin showed that the lysine residue at position 63 was required and sufficient for the generation of high-molecular weight ubiquitin conjugates, consistent with the K63 ubiquitin chain-specificity of the HECT domain. Ubiquitin variants containing one of the other lysine residues gave rise to a ubiquitylation pattern that resembled the pattern observed with Ub^K0^, suggesting that these ubiquitin variants only supported multi-monoubiquitylation (Fig. 1E). A similar pattern was observed for ubiquitylation of the ProxE3 with the only difference being that this was not dependent on administration of AP21967, in line with the idea that these modifications are due to autoubiquitylation (Fig. 1F). The formation of polyubiquitin chains with the HA-Ub^K63^ was also confirmed by probing the samples with an antibody specific for ubiquitin, confirming the nature of the high molecular weight products as ubiquitin conjugates (Fig. 1G). We conclude that recombinant ProxE3 specifically conjugates K63-linked ubiquitin chains to GFP-Sub in an AP21967-inducible manner in vitro.

### K48-linked ubiquitylation of ProxE3 in cells

**Figure 2:**
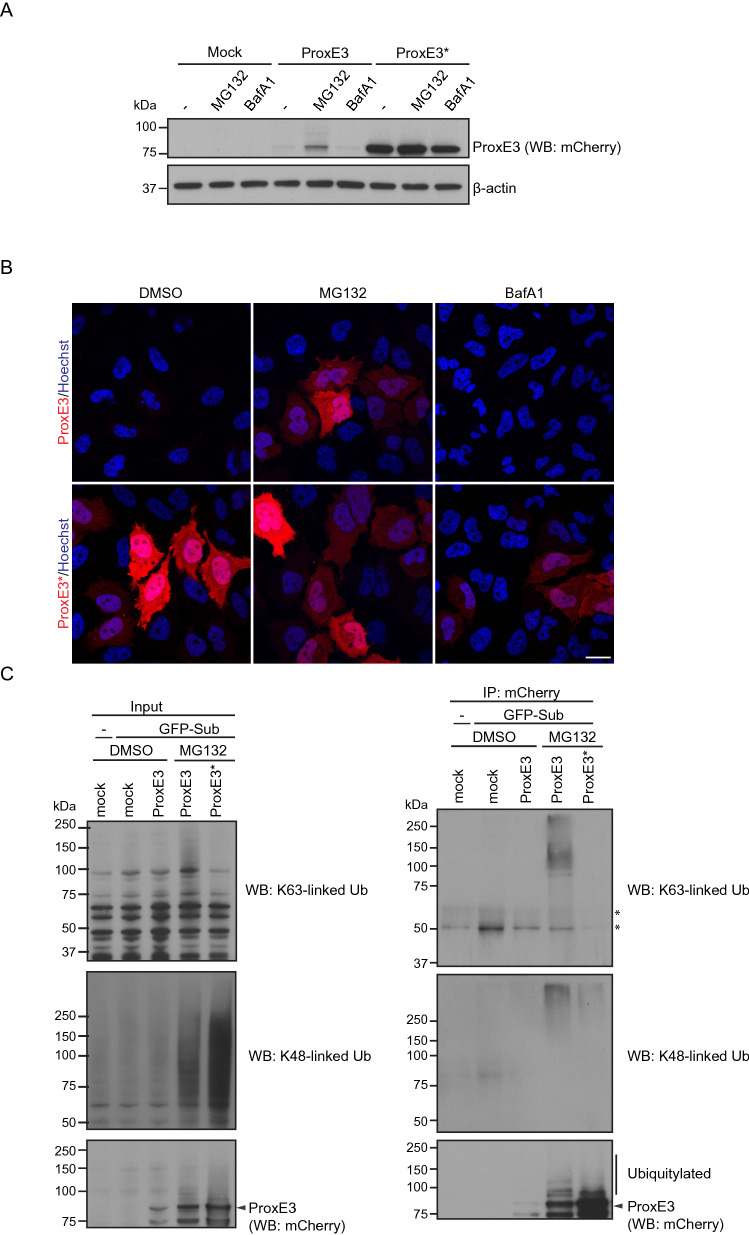
K48-linked ubiquitylation of ProxE3 in cells. (**A**) Western blot of HeLa cells transiently transfected with ProxE3, the catalytically inactive ProxE3* or mock transfected and treated for 5 h with 10 µM MG132, 100 nM Bafilomycin A1 (BafA1) or DMSO. (**B**) Representative confocal micrographs of HeLa cells transiently transfected with mCherry-ProxE3, -ProxE3* or mock transfected and treated for 8 h in the presence or absence of 10 µM MG132, 100 nM Bafilomycin A1 (BafA1) or DMSO. Hoechst counter staining is represented in blue. Scale bar: 20 µm. (**C**) Denaturing immunoprecipitation of mCherry-fusions in parental HeLa cells (−) or HeLa cells stably expressing GFP-Sub (GFP-Sub) and transfected with mCherry-tagged ProxE3, the catalytically inactive ProxE3* or mock transfected and treated for 12 h in the presence or absence of 10 µM MG132 (+ MG132 and − MG132 respectively). Arrows are pointing towards the unmodified protein of interest (either GFP-Sub or ProxE3), asterisks are indicating aspecific bands.

Next, we expressed the catalytically active ProxE3 and catalytically inactive ProxE3* in human cervical carcinoma HeLa cells. Surprisingly, we found that while we could readily detect the inactive ProxE3* by Western blotting (Fig. 2A), the steady-state levels of the active ProxE3 ligase were very low. Notably, treatment of cells with the proteasome inhibitor MG132, but not the autophagy inhibitor Bafilomycin A1, resulted in an increase in the ProxE3 levels (Fig. 2A), suggesting that rapid turnover by proteasomal degradation is responsible for the low levels of the ectopically expressed ubiquitin ligase. Confocal microscopy confirmed that the ProxE3 levels increased upon administration of proteasome inhibitor whereas Bafilomycin A1 had no effect (Fig. 2B).

This was unexpected considering that K63-linked ubiquitin chains do not target proteins for proteasomal degradation^3,4^. To probe into this question, we had a closer look at the ubiquitylation status of the engineered ubiquitin ligase. This experiment was performed in HeLa cells expressing GFP-Sub (Suppl. Fig. S1). mCherry-ProxE3 and mCherry-ProxE3* were immunoprecipated and precipitates were probed with antibodies specific for K63-linked or K48-linked ubiquitin chains, with the latter being a canonical signal for proteasomal degradation^2^. This revealed that, distinct from the in vitro reaction, in which we exclusively detected K63-linked ubiquitylation of ProxE3, the ligase was modified with both K63-linked and K48-linked ubiquitin chains when expressed in cells. These conjugates could only be detected upon stabilization of the protein with proteasome inhibitor and were dependent of the catalytic activity as ProxE3* was not modified by these ubiquitin chains (Fig. 2C). Thus, despite ProxE3 being a K63-specific ubiquitin ligase in vitro, the protein is additionally modified with K48-linked ubiquitin chains in cells, resulting in reduced steady-state levels due to proteasomal degradation. It is presently unclear whether the K48-linked ubiquitin chains are due to autoubiquitylation or the product of endogenous ubiquitin ligases that target ProxE3 dependent on its catalytic activity, which could be caused by K48-linked ubiquitin ligases modulating K63-linked ubiquitin chains^29^.

### ProxE3 conjugates K63-linked ubiquitin chains to mitochondria-tethered mitoGFP-Sub in cells

**Figure 3:**
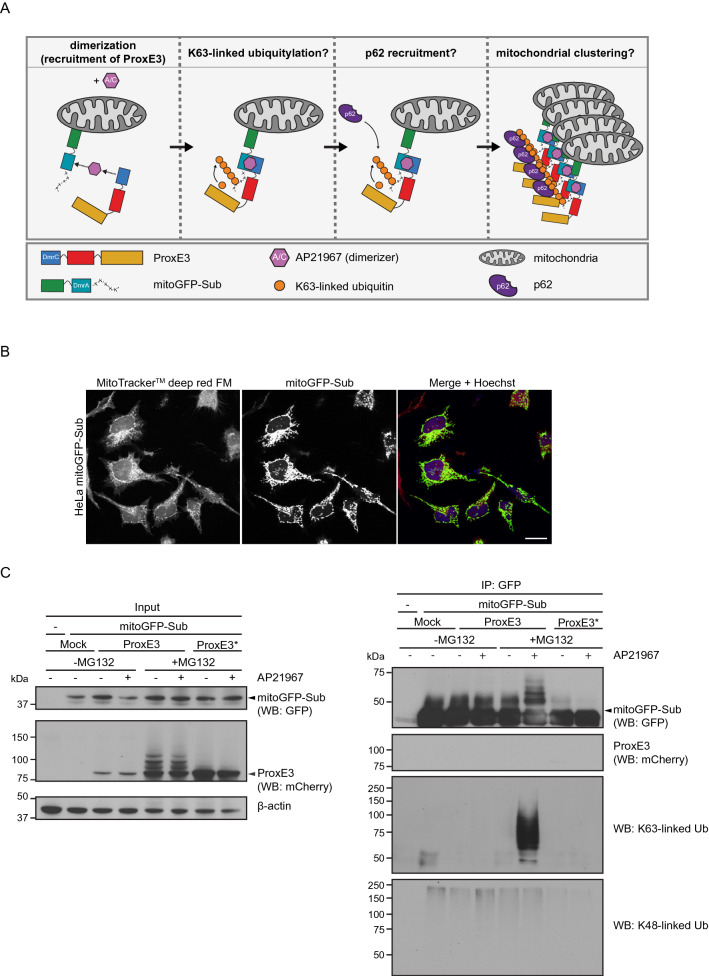
ProxE3 conjugates K63-linked ubiquitin chains to mitochondria-tethered mitoGFP-Sub in cells. (**A**) Schematic illustration of the inducible and K63-linkage specific ubiquitylation system comprised of ProxE3 and its mitochondria-localized substrate mitoGFP-Sub. (**B**) Representative confocal images of HeLa cells stably expressing mitoGFP-Sub. (**C**) Denaturing immunoprecipitation of GFP fusions in parental HeLa cells (−) or HeLa cells stably expressing mitoGFP-Sub (mitoGFP-Sub) and transiently transfected with mCherry-tagged ProxE3, the catalytically inactive ProxE3* or mock transfected and treated for 12 h in the presence or absence of 10 µM MG132 (+ MG132 and − MG132 respectively) and/or 400 nM AP21967. Arrows indicate the unmodified protein of interest (either mitoGFP-Sub or ProxE3).

To explore the applicability of ProxE3 as an inducible K63 ubiquitin ligase, we focused on the role of K63-linked ubiquitylation in mitochondrial sequestration and mitophagy. Parkin-mediated K63-linked ubiquitylation of MOM proteins on dysfunctional mitochondria is implicated in mitochondrial sequestration^11,12^. We reasoned that our model system should allow us to bypass recruitment of Parkin and generate K63-linked ubiquitin chains on mitochondria in the absence of mitochondrial uncoupling (Fig. 3A). To this end, we stably expressed in HeLa cells mitoGFP-Sub, which is a modified version of the above-mentioned substrate that contains the mitochondrial transmembrane domain of TOM20 to facilitate tethering to the mitochondrial surface. Indeed, this substrate localized at mitochondria as illustrated by the co-localization of the GFP signal with a mitochondrial staining (Fig. 3B). Administration of proteasome inhibitor (to stabilize the active ligase) and AP21967 (to induce dimerization) to cells expressing ProxE3 resulted in the presence of high molecular weight mitoGFP-Sub species that were identified as K63-linked ubiquitin chains (Fig. 3C). Importantly, we did not detect K48-linked ubiquitin chains on mitoGFP-Sub. K63-linked ubiquitin conjugates were only observed with catalytically active ProxE3, suggesting that its specific activity as a K63-specific ubiquitin ligase is well preserved in cells (Fig. 3C).

### K63-linked ubiquitin chains are sufficient to induce p62 recruitment and mitochondrial sequestration

**Figure 4:**
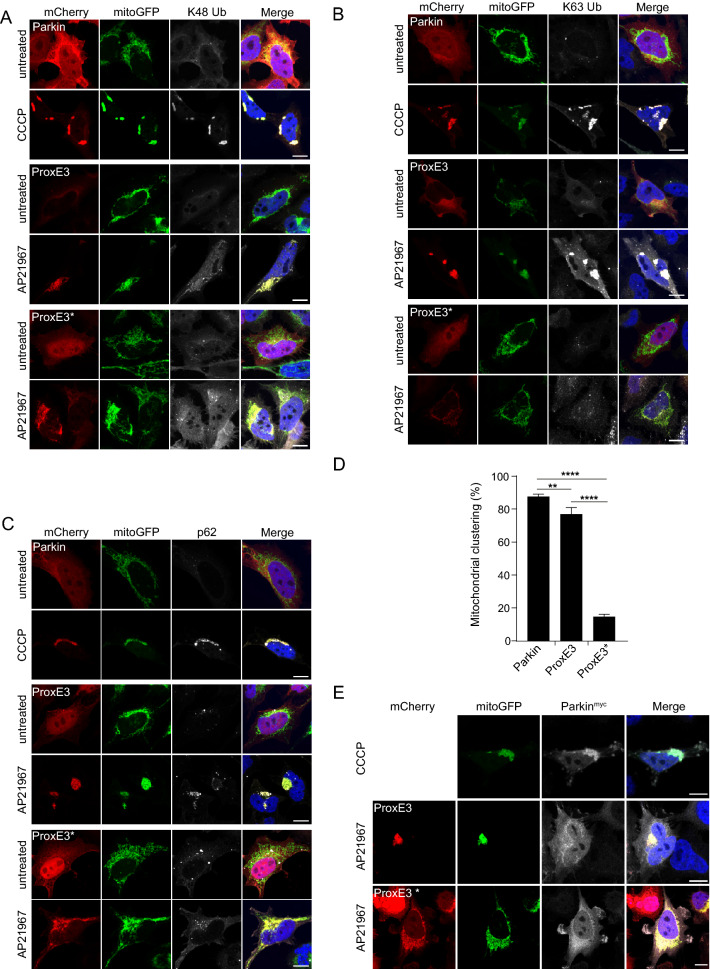
K63-linked ubiquitin chains are sufficient to induce p62 recruitment and mitochondrial sequestration. Representative confocal images of HeLa cells stably expressing mitoGFP-Sub, transfected with mCherry-tagged Parkin, ProxE3 or the catalytically inactive ProxE3* and treated for 16 h with 10 µM CCCP or 400 nM AP21967. Cells were not treated with proteasome inhibitor MG132. Cells were fixed and stained using a K48-linkage-specific ubiquitin antibody (**A)**, a K63-linkage specific ubiquitin antibody (**B**), an anti-p62 antibody (**C**). Magnified regions are indicated. Scale bar: 10 µm. (**D**) Quantification of the percentage of cells displaying perinuclear sequestration of their mitochondria. Data shown represent the mean ± SD of three independent experiments. For each experiment 100 cells were scored. ****p < 0.0001, ***p < 0.001, **p < 0.01 (one-way ANOVA, Sidak’s multiple comparison test). (**E**) Representative confocal images of HeLa cells stably expressing mitoGFP-Sub transiently transfected with Parkin^myc^ or co-transfected with Parkin^myc^ and mCherry-ProxE3 or catalytically inactive mCherry-ProxE3*. Cells were treated for 16 h with 10 µM CCCP or 400 nM AP21967 and stained using an anti-myc antibody. Scale bar: 10 µm.

In order to determine if K63-linked ubiquitin chains alone can facilitate sequestration of mitochondria, we generated a HeLa cell line stably expressing mitoGFP-Sub and analyzed the effect of AP21967-administration upon transient transfection of mCherry-ProxE3 or -ProxE3*. As a reference, we compared this with the effect of ectopic expression of mCherry-Parkin in the presence of the mitochondrial uncoupler carbonyl cyanide *m*-chlorophenyl hydrazine (CCCP) to inflict mitochondrial dysfunction. Administration of CCCP or AP21967 did not affect the distribution of mitochondria in mitoGFP-Sub-expressing cells in the absence of mCherry-Parkin or mCherry-ProxE3 (Suppl. Fig. S2). Consistent with the role of Parkin, mCherry-Parkin-expressing cells only displayed clustering of mitochondria when mitochondrial damage was induced by CCCP administration (Fig. 4A–D). However, administration of AP21967 to ProxE3-expressing cells induced dramatic changes in mitochondrial localization in the absence of the mitochondrial uncoupler (Fig. 4A–D). ProxE3, but not ProxE3*, expressing cells displayed a loss of the tubular organization of the mitochondrial network and sequestration of mitochondria in the perinuclear region, comparable to the CCCP-dependent effect of Parkin. Unlike CCCP administration to Parkin-expressing cells, ProxE3 expression and AP21967 administration had no effect on the viability of the cells (Suppl. Fig. S3). Expression of mCherry-Parkin resulted in robust K48-linked ubiquitylation of mitochondria in cells treated with CCCP, while K48-linked ubiquitin chains were almost absent on mitochondria in cells expressing ProxE3 (Fig. 4A). On the contrary, we observed that both ligand-induced tethering of ProxE3 and CCCP-induced translocation of mCherry-Parkin resulted in accumulation of K63-linked ubiquitin chains on the mitochondrial surface (Fig. 4B). This confirms that ProxE3 is a selective K63 ubiquitin ligase that shares the ability to label the mitochondrial surface with K63-linked ubiquitin chains with Parkin. The scaffold protein p62 binds K63-linked ubiquitin chains and has been shown to play a crucial role in Parkin-facilitated mitochondrial clustering in the perinuclear region^15,16^. Both in cells expressing mCherry-Parkin upon exposure to CCCP and in cells expressing ProxE3 upon administration of AP21967, we found that the clustered mitochondria were decorated with p62 (Fig. 4C). Quantitative analysis confirmed that the sequestration of CCCP-damaged and undamaged mitochondria was comparable in mCherry-Parkin- and ProxE3-expressing cells, respectively, but did not occur in cells expressing the catalytically inactive ProxE3* (Fig. 4D). While myc-tagged Parkin (Parkin^myc^) co-localized with mitochondria in CCCP-treated cells, it was not recruited to the sequestered mitochondria in cells expressing mCherry-ProxE3 and mitoGFP-Sub, suggesting that the functionality of these mitochondria is not compromised (Fig. 4E). Together, these data show that the generation of K63-linked ubiquitin chains on the mitochondrial surface is sufficient to induce p62-mediated sequestration in the absence of extrinsically inflicted mitochondrial damage.

### K63-linked ubiquitin chains are insufficient to induce mitophagy

**Figure 5:**
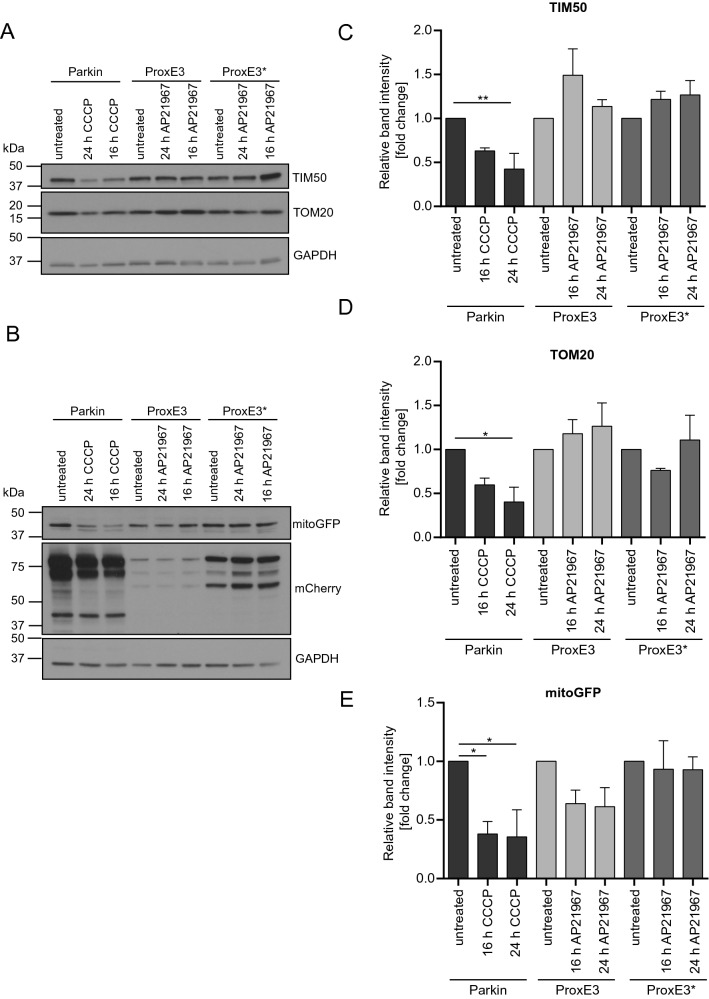
K63-linked ubiquitin chains are insufficient to induce mitophagy. (**A**, **B**) Representative Western blots of whole cell lysates of HeLa cells stably expressing mitoGFP-Sub transiently transfected with mCherry-Parkin, mCherry-ProxE3 or mCherry-ProxE3*. Cells were either left untreated or were treated for 16 h or 24 h with 10 µM CCCP or 400 nM AP21967. Quantification of the relative band intensities for TIM50 (**C**), TOM20 (**D**) and mitoGFP-Sub (**E**). Data were normalized to the loading control GAPDH and are expressed as fold change compared to untreated cells. Data shown represent the mean ± SEM of three (TOM20, mitoGFP-Sub) or four (TIM50) independent experiments. **p < 0.01, *p < 0.05 (one-way ANOVA, Sidak’s multiple comparison test).

To analyze if mCherry-ProxE3-mediated ubiquitylation of mitochondria also induced mitophagy, we compared the levels of the mitochondrial outer membrane protein TOM20, the mitochondrial inner membrane protein TIM50 (Fig. 5A) as well as the engineered substrate mitoGFP-Sub (Fig. 5B) in ProxE3-, ProxE3*- and Parkin-expressing cells. Semiquantitative analysis by Western blotting showed that CCCP-treated, Parkin-expressing cells but not AP21967-treated, ProxE3- or ProxE3*-expressing cells, displayed a reduction in the levels of TIM50 (Fig. 5C) and TOM20 (Fig. 5D). The levels of the engineered substrate mitoGFP-Sub were clearly reduced in Parkin-expressing cells upon administration of CCCP (Fig. 5E). A reduction in mito-GFP-Sub was also found in ProxE3-expressing cells although not to the same extent as in cells expressing Parkin (Fig. 5E). The lack of degradation of endogenous mitochondrial outer and inner membrane proteins in ProxE3-expressing cells suggests that K63-linked ubiquitylation of mitoGFP-Sub is not sufficient to target mitochondria for mitophagy.

## Discussion

Here we investigated the proficiency of K63-linked polyubiquitin chains to target mitochondria for sequestration and autophagy. For this purpose, we developed a ubiquitin ligase that decorated an inherent reference protein located at the outer membrane of mitochondria with K63-linked polyubiquitin chains. Interestingly, the presence of these ubiquitin chains dramatically altered the localization of the mitochondria in the absence of extrinsically inflicted damage, resulting in sequestration of mitochondria in the perinuclear region, a process that has been shown to be linked to K63-linked ubiquitylation and p62 during Parkin-mediated mitophagy^15,16^.

The role of ubiquitin chains in targeting misfolded proteins to inclusion bodies or autophagosomes is not well understood. While it is clear that both processes involve specific ubiquitin receptors, it has been suggested that protein oligomerization instead of ubiquitylation, is the central driver in this process^7^. Consistent with this idea, early findings already suggested that misfolded proteins can form inclusion bodies without being subject to ubiquitylation^30^. On the other hand, ubiquitin can function as an autonomous signal in autophagy as a membrane-tethered, lysine-less ubiquitin mutant was shown to be able to target peroxisomes for lysosomal degradation^31^. While this paper was under review, it was reported that short linear ubiquitin chains composed of lysine-less ubiquitin, when tethered to mitochondria, are sufficient to induce mitophagy, while wild-type ubiquitin chains fail to do so^14^. These data suggest that in these artificial systems the absence of lysine residues in ubiquitin, which will among others prevent the formation of branched ubiquitin chains, may be a critical determinant to target organelles for lysosomal degradation. Consistent with this study, our findings show that K63-linked ubiquitylation of mitochondrial proteins does not lead to autophagic degradation while still being able to recruit the ubiquitin receptor p62 and induce mitochondrial sequestration.

For functional mitophagy, fragmentation of mitochondria, which occurs in Parkin-expressing cells, has also been suggested to be required to obtain small enough entities that can be handled by the autophagy machinery^15,32^. The mode of action of Parkin itself is more complex, resulting in the synthesis of additional chains, such as K6-, K27- and K48-linked ubiquitin chains, which may provide additional signals that target for mitophagy^9,23,33^. Several studies support a critical role of Parkin-induced proteasomal degradation of MOM proteins in autophagic targeting, likely by induction of membrane rupture and exposure of proteins on the inner mitochondrial membrane that serve as specific mitophagy receptors^24,25^. It is therefore tempting to speculate that sequestration of mitochondria may be the default outcome for mitochondria decorated with K63-linked ubiquitin chains whereas additional modifications may be required to change the fate of mitochondria from sequestration to lysosomal degradation. The nature of these additional cues may function as a fail-safe mechanism to prevent the irreversible elimination of proper functioning mitochondria.

## Materials and methods

### Plasmids

ProxE3 expression plasmid was generated by PCR amplifying the HECT domain of the pGEX6P1-NEDD4 plasmid (a gift from Simona Polo) and inserting it into a pmCherry-C1 (Clontech) expression vector via *Bsr*GI and *Not*I restriction sites. Next, the dimerization domain (DmrC) was introduced by PCR amplifying the DmrC sequence from the pHet-1 (Takara Bio USA) plasmid and assembled using *Sac*II and *Bam*HI restriction sites. The catalytic dead ProxE3 was generated by hybridizing oligonucleotides (5′-GCC AAG AGC TCA TAC CTC TTT TAAT-3′) containing a mutation changing the catalytic cysteine for a serine in the C-terminal HECT domain of NEDD4-1. The fragment was then digested by *Sac*I/*Eco*RI and inserted in the pGEX6P1-NEDD4 plasmid using *Sac*I and *Eco*RI restriction sites. The NEDD4* fragment from pGEX6P1-NEDD4* was subsequently inserted into the DmrC-mCherry-NEDD4 plasmid using the *Bsr*GI and *Not*I restriction sites. The plasmid coding for GFP reporter substrate was generated by PCR amplifying the DmrA domain from pHet-Nuc1 plasmid (Takara Bio USA) and inserting it into a pEGFP-C1 expression vector via the *Bgl*II and *Eco*RI restriction sites. The lysine tail was then generated by hybridizing complementary oligonucleotides (sense: 5′-GAC CGA ATT CAA CAA TAA GAA TAA CAA GAA CAA CAA GAA TAA TAA GAA TAA CAA GTG ACC GCG GAC-3′) and insertion into the GFP reporter substrate plasmid via the *Eco*RI and *Sac*II restriction sites. The mitochondrial GFP reporter plasmid was generated by PCR amplifying the first 99 nucleotides of TOM20 coding for its mitochondrial intermembrane and transmembrane domain from the pcDNA3-mitoCKAR plasmid^34^ (gift from A. Newton) and inserted it into the GFP reporter plasmid via the *Nhe*I and *Age*I restriction sites. The mito-GFP-DmrA-Ktail sequence was then excised from the pEGFP-C1 backbone and inserted into a pCMV-IRES-puro vector via the *Nhe*1 and *Not*I restriction sites. HA-ubiquitin plasmid was generated by hybridizing oligonucleotides coding for HA and inserting them into the ubiquitin coding plasmid pEGFP-Ub^35^ using the *Age*I and *Bgl*II restriction sites. The ProxE3 and GFP reporter substrate coding sequence was inserted into bacterial expression vectors pGEX-6P-1 via the *Sal*I and *Not*I restriction sites for production of GST tagged recombinant proteins. Ubiquitin single lysine only mutants were a gift from Simona Polo. All mock transfections were carried out with empty pGEX6P1 vectors (Clontech). All constructs were verified by DNA sequencing.

### Cell lines

HeLa and HeLa Flp-In^TM^ T-REx^TM^ cells (Thermo Fisher Scientific) were cultured in Dulbecco's modified Eagle's medium (Invitrogen), supplemented with 10% fetal calf serum. Cells were transfected with plasmid DNA using Lipofectamine 2000 or Lipofectamine 3000 (Invitrogen) according to the manufacturer's instructions. Cell lines stably expressing the soluble and mitochondrial GFP reporter substrates were generated by transfecting HeLa Flp-In T-REx cells with pCMV-IRES-puro-GFP-DmrA-Ktail and pCMV-IRES-puro-mito-GFP-DmrA-Ktail respectively and selecting cells with stably integrated plasmids in medium containing 400 μg/ml Zeocin, 5 μg/ml Blasticidin S and 1 μg/ml Puromycin (Sigma). The stable cell-line expressing the mitochondrial reporter substrate was then FACS sorted based on high level of expression. Cells were analyzed 24 h after transfection. All cell lines were tested for mycoplasma contamination. Cells were treated with variable concentrations of AP21967 (heterodimerizer A/C; Takara Bio USA) as indicated in the respective figure legends, 10 μM CCCP (Sigma-Aldrich), 10 μM MG132 (Enzo), 100 nM Bafilomycin A1 (Enzo) or DMSO (Sigma-Aldrich). Mitochondria were stained with 200 nM MitoTracker Deep Red FM (Thermo Fisher Scientific).

### Protein purification

Wild type and mutant ubiquitin were expressed in Rosetta(DE3)pLysS cells and purified using an adapted protocol^36^. Bacteria were suspended in low salt buffer (25 mM ammonium acetate, 25 mM NaCl, 10 mM 2-mercaptoethanol, 10% glycerol, and proteases inhibitor) at pH 7.0, and lysed by sonication. Cell debris was then removed by centrifugation and the supernatant adjusted to pH 4.5–5.0 with concentrated acetic acid. Acid-precipitable proteins were removed by centrifugation, and the supernatant was passed through a 0.45 μm CA filter (Corning). Dialysis was then performed overnight at 4 °C in 3.5 Kd MWCO tubes against dialysis buffer (50 mM Tris HCl, 100 mM NaCl, 10% glycerol, 1 mM EDTA) at pH 8.0. Ubiquitin was concentrated after dialysis using centricon 3.0 Kd MWCO (Sigma). Purity of proteins was assessed on Coomassie stained sodium dodecyl sulphate polyacrylamide gel electrophoresis (SDS-PAGE) gels. Ubiquitin preparation concentration was determined by Bradford Assay (Biorad). GST-tagged ProxE3, ProxE3* and GFP-substrate were expressed in BL21(DE3). Expression was induced by 0.2 mM IPTG at 18 °C for 16 h. Bacteria were lysed in GST lysis buffer (50 mM Hepes pH 7.5, 500 mM NaCl, 10% glycerol, 1 mM MgCl2, 10 mM DTT) by sonication. Cell debris was removed by centrifugation and the supernatant incubated on glutathione sepharose beads at 4 °C for 16 h. Beads were washed with GST lysis buffer and subsequently used as bead-immobilized GST-GFP-Sub. To obtain untagged, recombinant protein, beads were washed in PreScission cleavage buffer (50 mM Tris–Cl pH 7.5, 150 mM NaCl, 1 mM EDTA and 1 mM DTT) and untagged, recombinant protein then cleaved off the beads by 4 h of incubation with PreScission Protease (GE Healthcare Life Sciences) at 4 °C. Purity of proteins was assessed on Coomassie stained SDS-PAGE gels. Protein concentration was determined with a NanoDrop 2000 (Thermo Fisher Scientific) by measuring the absorbance at 280 nm.

### In vitro ubiquitylation assay

Pre-dimerization was carried out in ubiquitylation buffer (25 mM Tris–HCl pH 7.6, 5 mM MgCl_2_, 100 mM NaCl, 2 μM DTT) at 37 °C with 0.2 μM of ProxE3/ProxE3* and GFP substrate and 0.5 μM A/C heterodimerizer (Takara Bio USA). The ubiquitylation reaction was carried out at 37 °C for 1 h by adding 200 nM UbE1, 1 μM UbcH5C, 1.2 μM untagged or HA tagged (Boston Biochem) ubiquitin or K only ubiquitin and 2 mM ATP to the pre-dimerization mix. The reaction was stopped by adding one volume of 2× SDS-PAGE sample buffer (125 mM Tris/Cl pH 6.8, 20% glycerol, 4% SDS, 10 mM DTT and 0.02% bromophenol blue) and boiling at 95 °C for 10 min.

### Immunoprecipitations

For native immunoprecipitation, cells were lysed in co-IP lysis buffer (10 mM Tris/-HCl pH 7.5; 150 mM NaCl; 0.5 mM EDTA; 0.5% NP-40) supplemented with protease inhibitor cocktail. Cleared lysates were used for GFP- or mRFP-immunoprecipitation using GFP- or mRFP-TrapA beads (Chromotek), according to the manufacturer’s specifications. Elution was done by boiling for 10 min at 95 °C in 2× SDS-PAGE sample buffer. For denaturing immunoprecipitations, cells were lysed in denaturing lysis buffer (10 mM Tris/Cl pH 7.5; 150 mM NaCl; 0.5 mM EDTA; 1% SDS) and later diluted in co-IP lysis buffer without detergent.

### Western blotting and antibodies

Cell extracts were generated by cell lysis, boiled in 2× Laemmli sample buffer, separated by SDS–PAGE on 4–12% (Novex, Invitrogen) or 8% polyacrylamide with stacking gels, transferred to nitrocellulose (GE Healthcare Life Sciences) or PVDF membranes (Millipore) and probed with relevant primary antibodies. Western blots were visualized using either secondary antibodies coupled to peroxidase and ECL chemiluminescent substrate (GE Healthcare Life Sciences) followed by detection on X-ray films or, alternatively, using antibodies coupled to near-infrared fluorophores (LiCOR) followed by analysis with the Odyssey Infrared Imaging System (LiCOR). The following primary antibodies were used: mCherry (6G6) from Chromotek; GFP (ab290) from Abcam; p62 (BD610832) from BD Bioscience; β-actin (A2228) from Sigma-Aldrich; HA (16B12, MMS-101R) from Biolegend; ubiquitin (Z0458) from DAKO; ubiquitin Lys-48 (Apu2, 05-1307) and ubiquitin Lys-63 (Apu3, 05-1308) from Millipore; TOM20 (FL-145, sc-11415) and TIM50 (C-9, sc-393678) from Santa Cruz Biotechnologies; and GAPDH (ab9485), from Abcam.

### Immunocytochemistry

HeLa cells stably expressing the mitochondrial reporter substrate were transiently transfected with mCherry-Parkin, mCherry-ProxE3 or mCherry-ProxE3*. Seven hours post transfection, cells were treated with 10 µM CCCP or 400 nM heterodimerzier A/C for 16 h. Cells were fixed in 4% paraformaldehyde (PFA) for 15 min. Permeabilization was done using 0.2% Triton X-100 for 5 min, followed by quenching with 100 mM glycine. Blocking was done in 3% BSA and primary antibodies were diluted in 0.1% Tween-20 in PBS and incubated for 2 h at RT. Secondary antibodies were diluted in 0.1% Tween-20 in PBS and incubated for 1 h at RT. Hoechst staining (Hoechst 33342, Thermo Fisher Scientific) in PBS was performed followed by mounting using homemade Mowiol/DABCO (Sigma) mounting medium. The following antibodies were used for immunocytochemistry: mouse anti-c-Myc 1:100 (9E10, sc-40, Santa Cruz Biotechnology), rabbit anti-p62 1:100 (H-290, sc-35575, SantaCruz Biotechnology), rabbit anti-ubiquitin Lys-48 1:100 (Apu2, 05-1307, Millipore) and ubiquitin Lys-63 1:100 (Apu3, 05-1308, Millipore).

### Microscopy

Confocal images were acquired using a Zeiss LSM510 META or LSM880 (Carl Zeiss) confocal laser scanning microscope equipped with Plan Apochromat 63×/1.40 DIC oil immersion objectives and LSM Imaging software in multi-track mode or Zen Black v2.1, respectively. Unless immunocytochemical stainings were performed, cells were fixed in 4% PFA (Sigma-Aldrich), counterstained with 2 ug/ml  Hoechst 33342 (Thermo Fisher Scientific) and mounted on glass slides (Marienfeld) using homemade Mowiol/DABCO medium (Sigma) mounting.

### Viability assay

HeLa cells stably expressing mitoGFP-Sub substrate were left untransfected or were transiently transfected with mCherry-Parkin, mCherry-ProxE3 or mCherry-ProxE3*. Seven hours post transfection, cells were either treated for 16 h with 400 nM AP21967, 10 µM CCCP or were left untreated. Cells were washed in PBS, fixed in 4% PFA (Sigma-Aldrich) for 15 min and incubated in 2 µg/ml Hoechst (Hoechst 33342, Thermo Fisher Scientific) in PBS for 15 min. Widefield fluorescent images were acquired using the ImageXpress Micro XLS microscope (Molecular Devices) equipped with a 20× objective. Nuclei were counted using the MetaXpress software (Molecular Devices).

### Assessment of functionality of mitochondria

HeLa cells stably expressing the mitoGFP-Sub were transiently transfected with Parkin^myc^ or co-transfected with Parkin^myc^ and mCherry-ProxE3 or mCherry-ProxE3*. Seven hours post transfection, cells were treated for 16 h with 10 µM CCCP or 400 nM heterodimerizer AP21967. Cells were fixed in 4% PFA for 15 min, and immunocytochemistry using anti-c-Myc antibody (9E10, sc-40, Santa Cruz Biotechnology) was performed.

### Statistical analysis

Statistical analysis was performed using GraphPad Prism 6. Statistical significance was tested using one-way ANOVA followed by Sidak’s multiple comparison test. The applied statistical tests are indicated in the respective figure legend. Data are presented as mean ± standard deviation (SD) or mean ± standard error of the mean (SEM), as indicated in the respective figure legend. Quantification of immunoblots was done with ImageJ 1.48v or Image Studio 4.0 software. Data are presented as fold change compared to control. The following p-values were considered significant: *p < 0.05, **p < 0.01, ***p < 0.001, ****p < 0.0001.

## Supplementary Information

Supplementary Information.
