# Supplementary material for: K63-linked ubiquitylation induces global sequestration of mitochondria
Source: Sci Rep. 2020 Dec 18;10:22334. doi: 10.1038/s41598-020-78845-7 (PMC7749161; doi:10.1038/s41598-020-78845-7)
Supplement: Supplementary file 1 — Supplementary Information. [file 41598_2020_78845_MOESM1_ESM.pdf]

Supplementary Information

K63-linked ubiquitylation induces global sequestration of mitochondria

Thibaud J.C. Richard, Laura K. Herzog, Julia Vornberger, Aldwin Suryo Rahmanto, Olle Sangfelt, Florian A. Salomons and Nico P. Dantuma

Content

- Supplementary Figure Legends
- Supplementarty Figures
- Uncropped blots

Suppl. Figure S1: Localization of GFP-Sub. Confocal micrograph of Hela cells stably expressing GFP-Sub. Scale bar = 20 μm.

Suppl. Figure S2: AP21867-treatment does not affect the subcellular localization of mitoGFP-Sub Representative confocal images of HeLa cells stably expressing mitoGFP-Sub. Cells were either left untreated or were treated for 16h with 10 μM CCCP or 400 nM AP21867. Scale bar = 10 μm.

Suppl. Figure S3: Expression of mCherry-ProxE3 and dimerization with its substrate mitoGFP-Sub does not affect cell viability. HeLa cells stably expressing mitoGFP-Sub and transiently transfected with. mCherry-Parkin, mCherry-ProxE3 or the catalytically inactive mCherry-ProxE3* were either left untreated or treated with 10 μM CCCP or 400 nM AP21967 for 16h and cell viability was determined by nuclei count. Data shown are expressed as percentage of cells compared to the untransfected and untreated control. Data are derived from two independent experiments.

Supplementary Figure S1

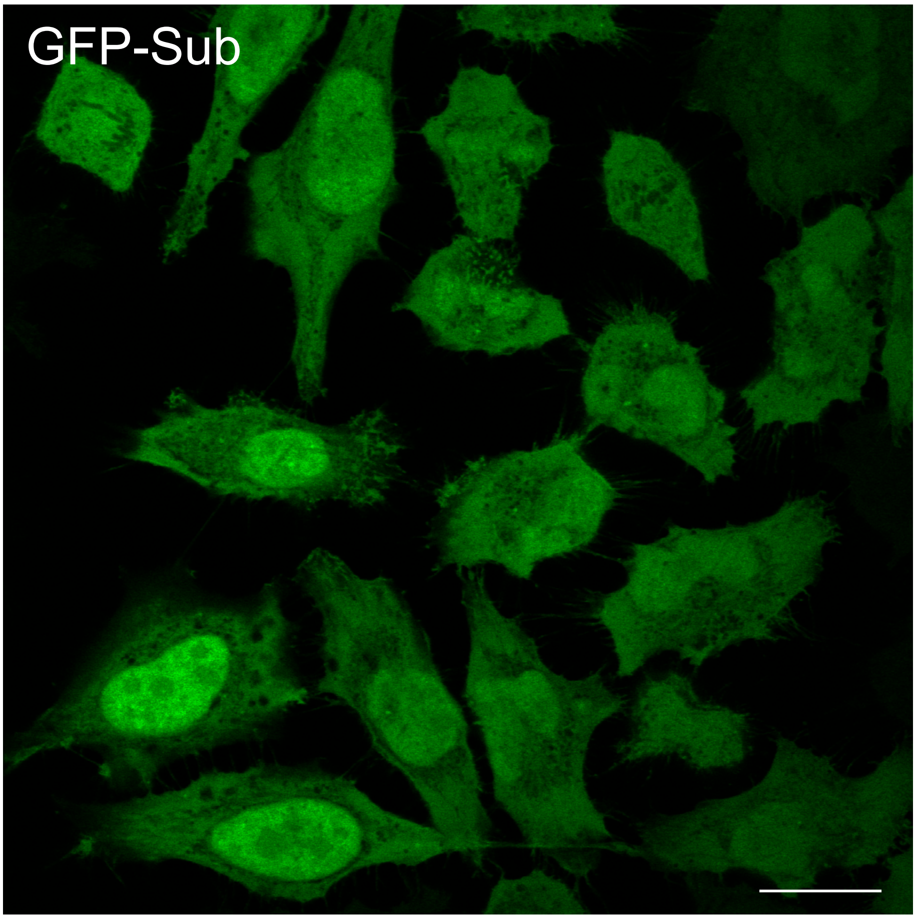

Supplementary Figure S2

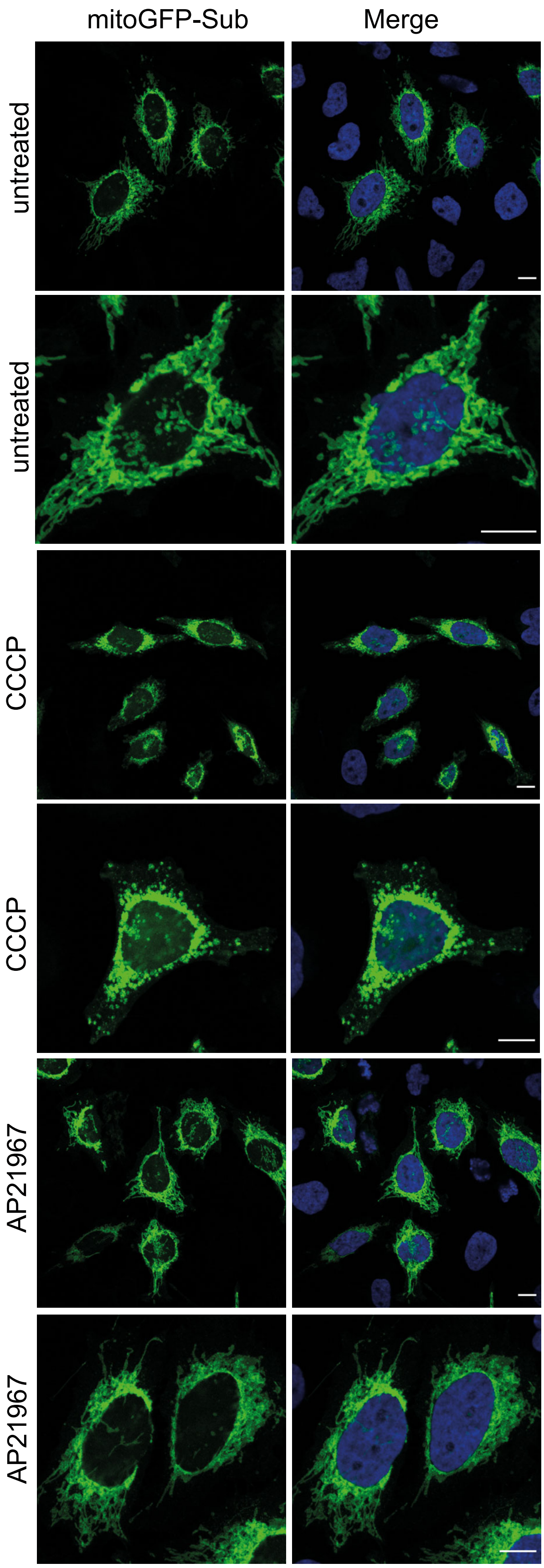

Supplementary Figure S3

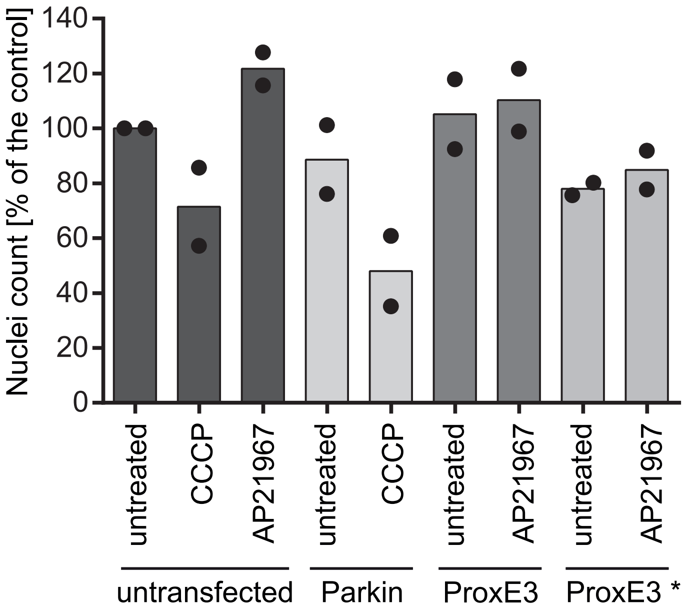

Figure 1 uncropped, unedited

B

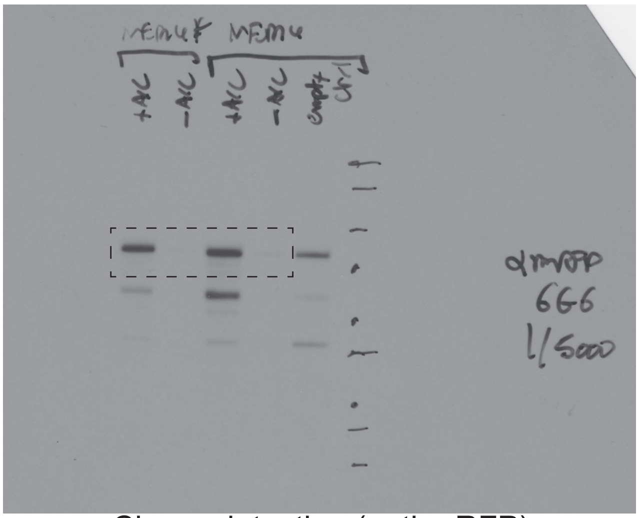

mCherry detection (anti-mRFP)
[mirrored in figure]

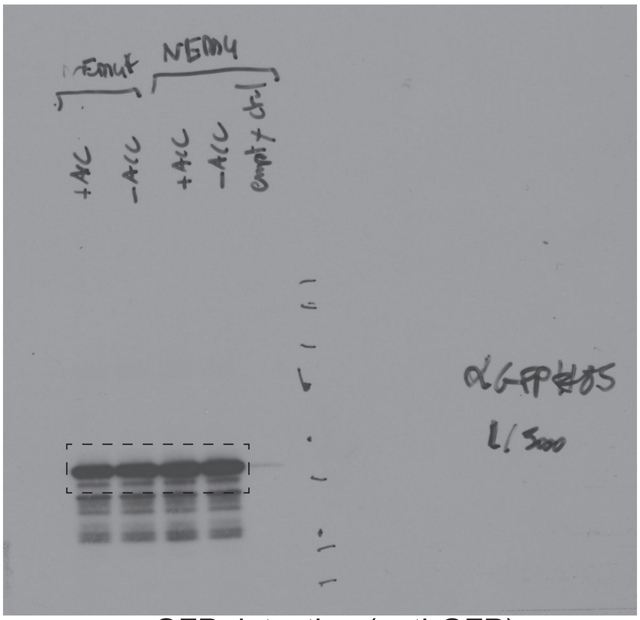

GFP detection (anti-GFP)
[mirrored in figure]

C

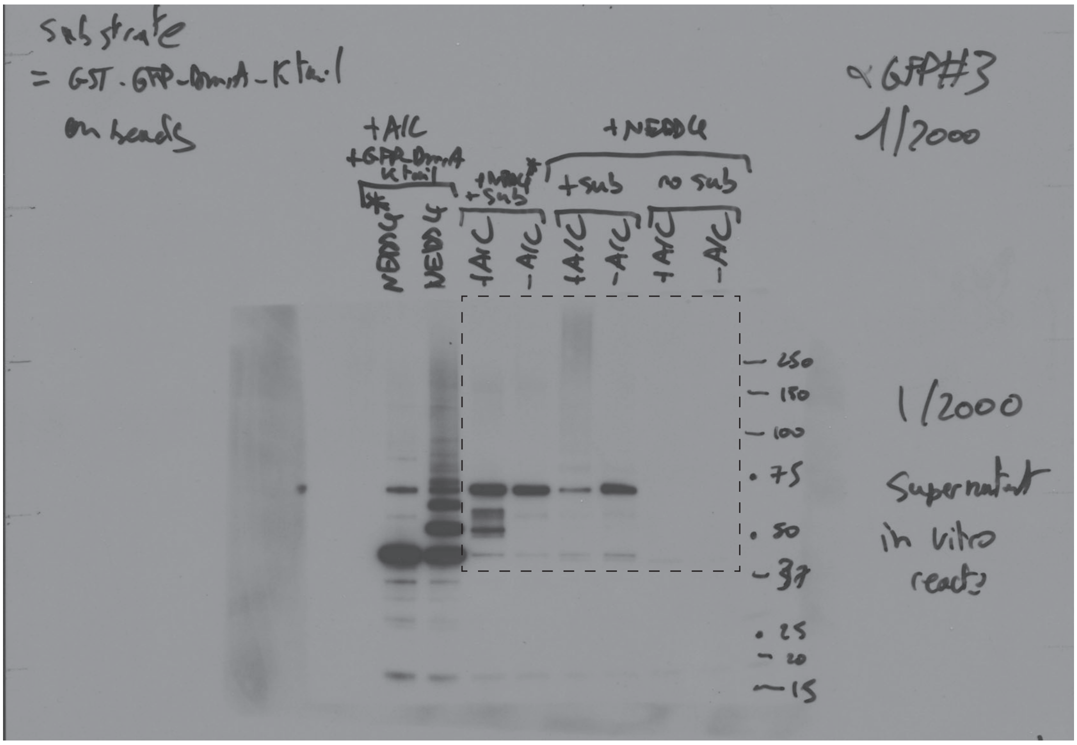

GFP detection (anti-GFP)
[mirrored in figure]

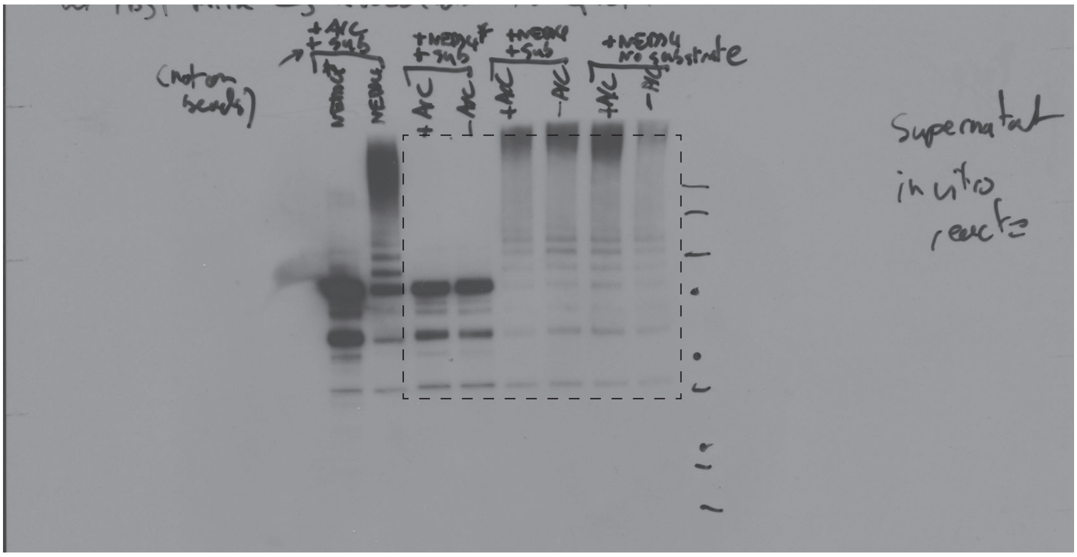

mCherry detection (anti-mRFP)
[mirrored in figure]

Figure 1 uncropped, unedited

D

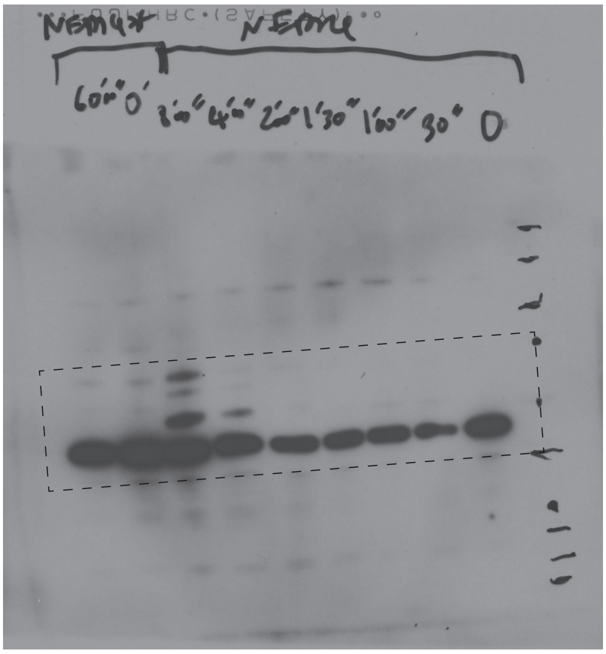

GFP detection (anti-GFP)
[mirrored in figure]

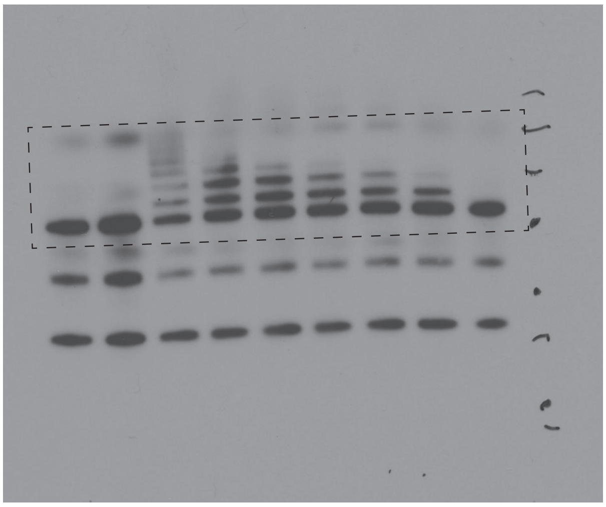

mCherry detection (anti-mRFP)
[mirrored in figure]

E

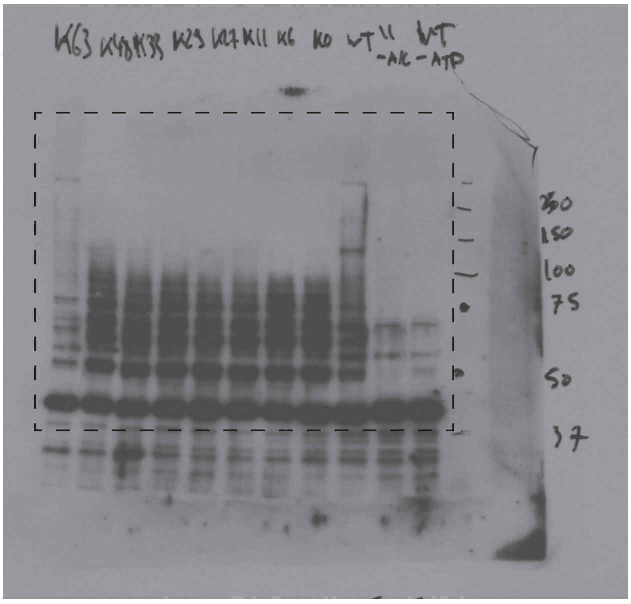

GFP detection (anti-GFP)
[mirrored in figure]

F

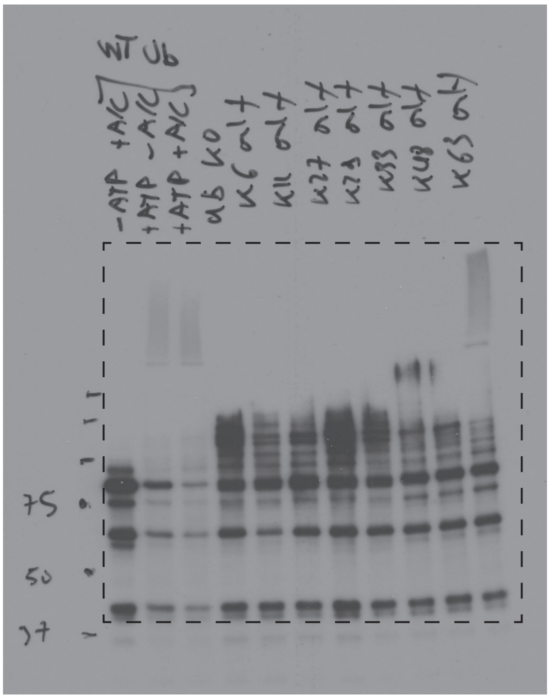

mCherry detection (anti-mRFP)
[mirrored in figure]

G

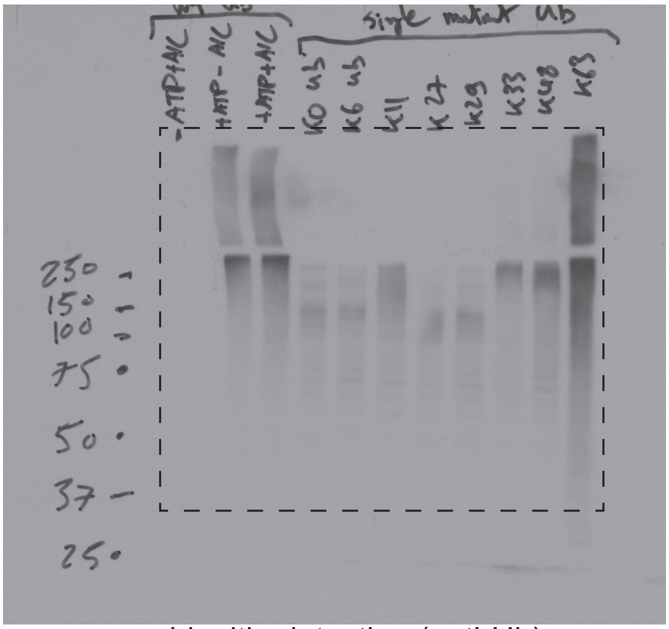

ubiquitin detection (anti-Ub)
[mirrored in figure]

Figure 2 uncropped, unedited

A

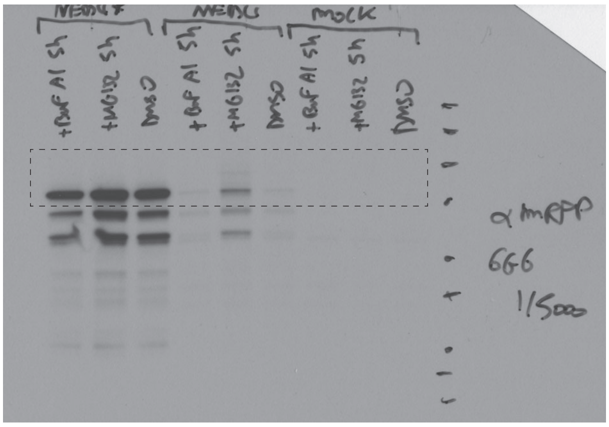

mCherry detection (anti-mRFP)
[mirrored in figure]

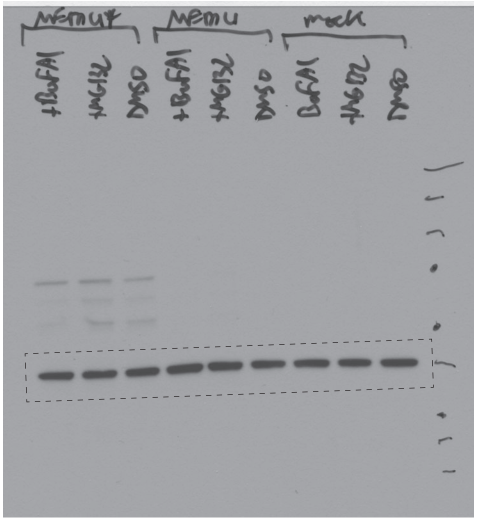

β-actin detection (anti-β-actin)
[mirrored in figure]

C

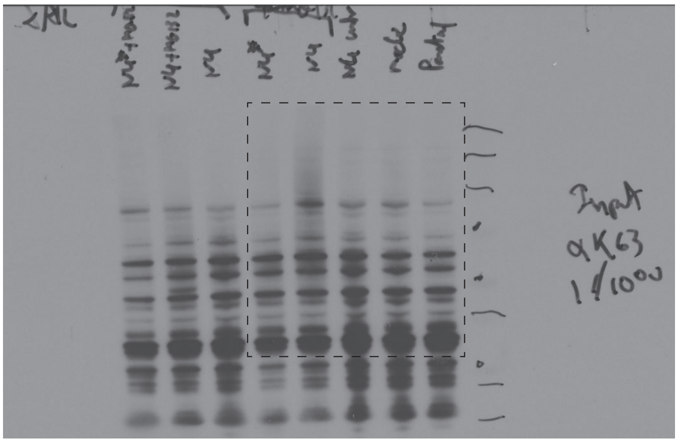

Input:
K63 detection (anti-K63-linked Ub)
[mirrored in figure]

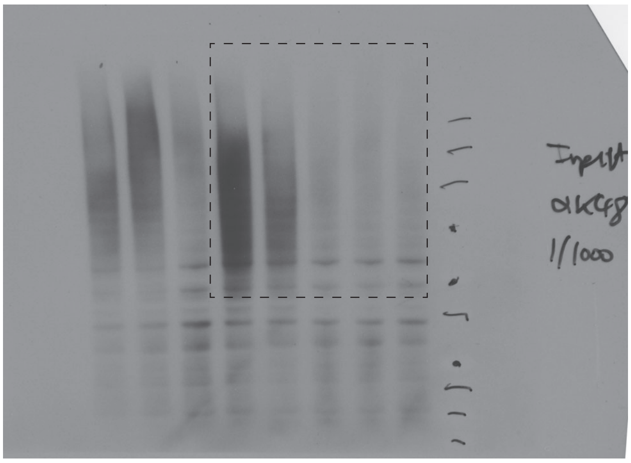

Input:
K48 detection (anti-K48-linked Ub)
[mirrored in figure]

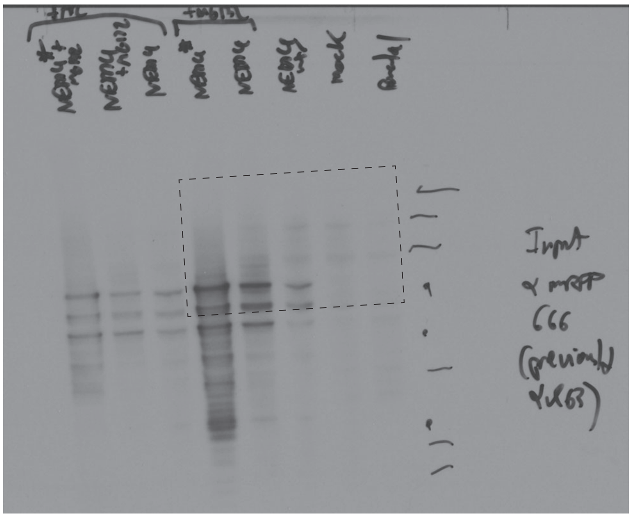

Input:
mCherry detection (anti-mRFP)
[mirrored in figure]

Figure 2 uncropped, unedited

C (continued)

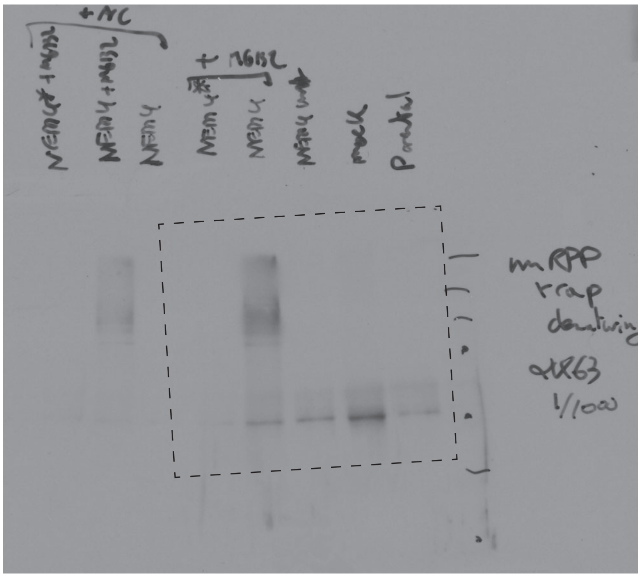

mRFP-Trap eluate:
K63 detection (anti-K63-linked Ub)
[mirrored in figure]

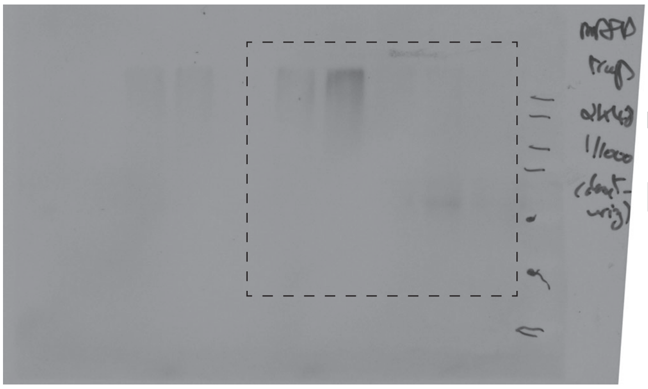

mRFP-Trap eluate:
K48 detection (anti-K48-linked Ub)
[mirrored in figure]

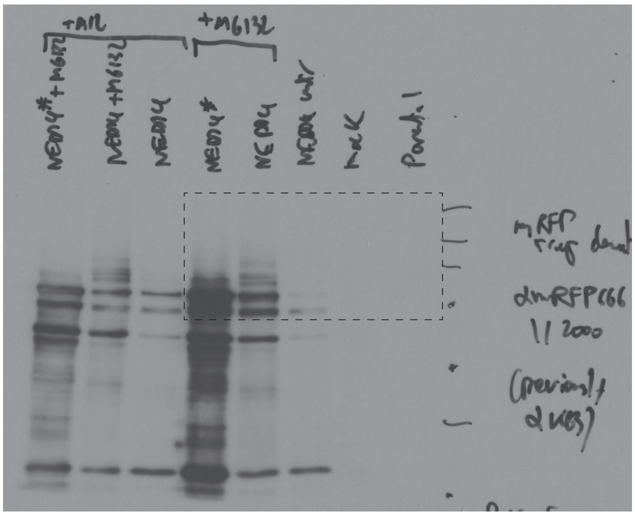

mRFP-Trap eluate
mCherry detection (anti-mRFP)
[mirrored in figure]

Figure 3 uncropped, unedited

C

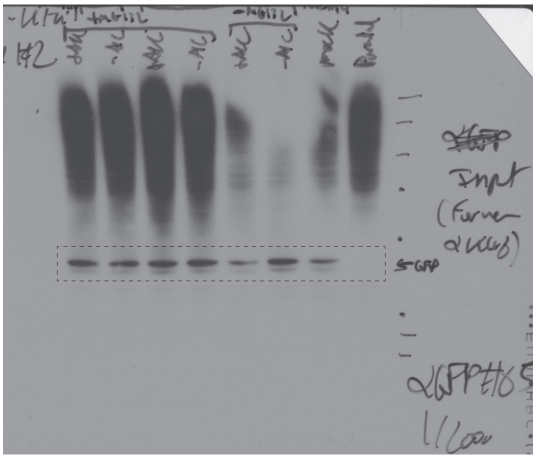

Input:
GFP detection (anti-GFP)
[mirrored in figure]

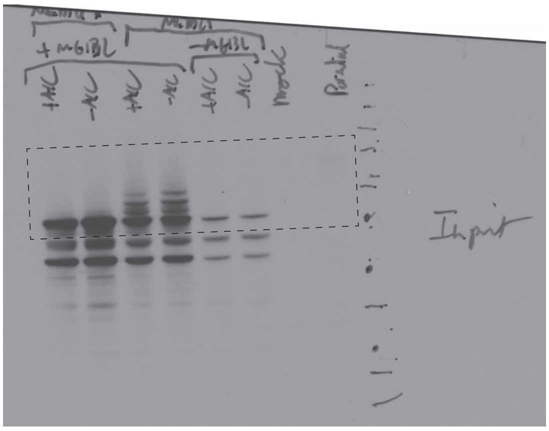

Input:
mCherry detection (anti-mRFP)
[mirrored in figure]

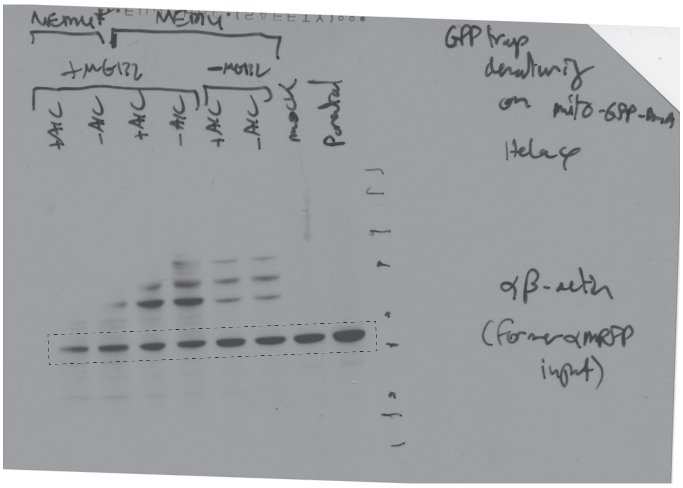

Input:
β-actin detection (anti-β-actin)
[mirrored in figure]

Figure 3 uncropped, unedited

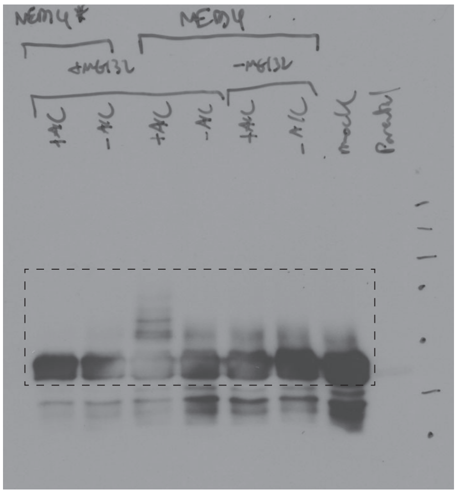

GFP-Trap eluate:
GFP detection (anti-GFP)
[mirrored in figure]

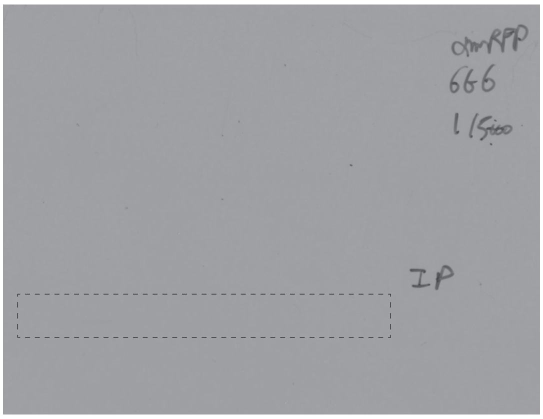

GFP-Trap eluate:
mRFP detection (anti-mRFP)
[mirrored in figure]

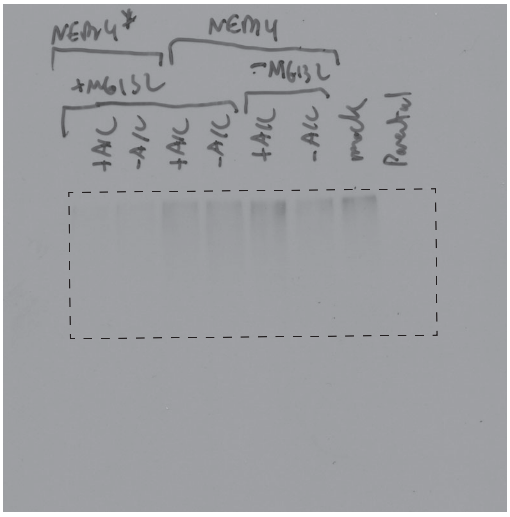

GFP-Trap eluate:
K48 detection (anti-K48-linked Ub)
[mirrored in figure]

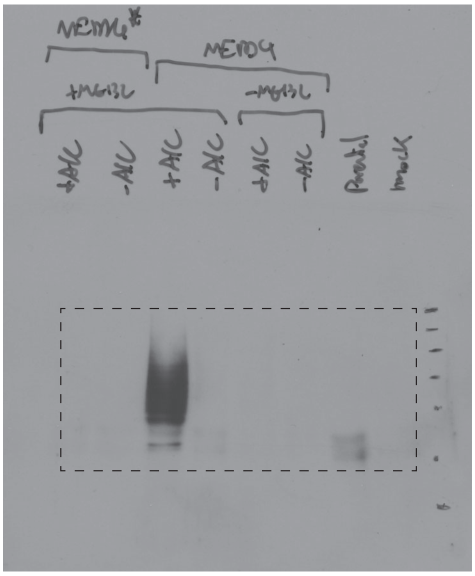

GFP-Trap eluate:
K63 detection (anti-K63-linked Ub)
[mirrored in figure]

Figure 5 uncropped, unedited

A

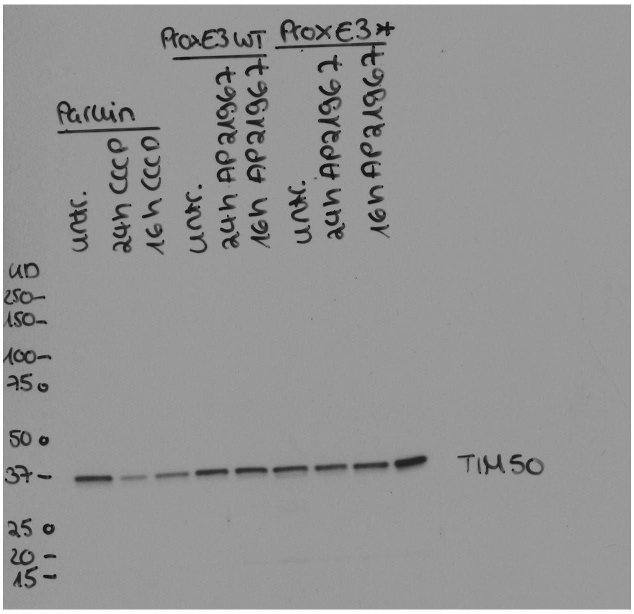

TIM50 detection (anti-TIM50)

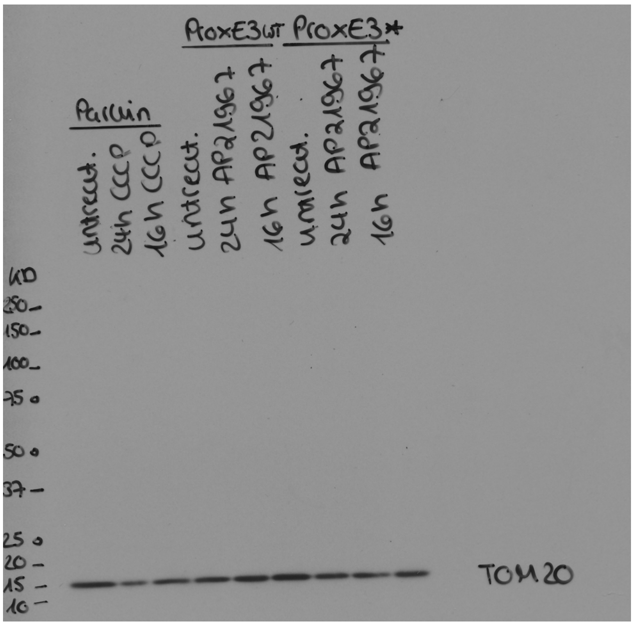

TOM20 detection (anti-TOM20)

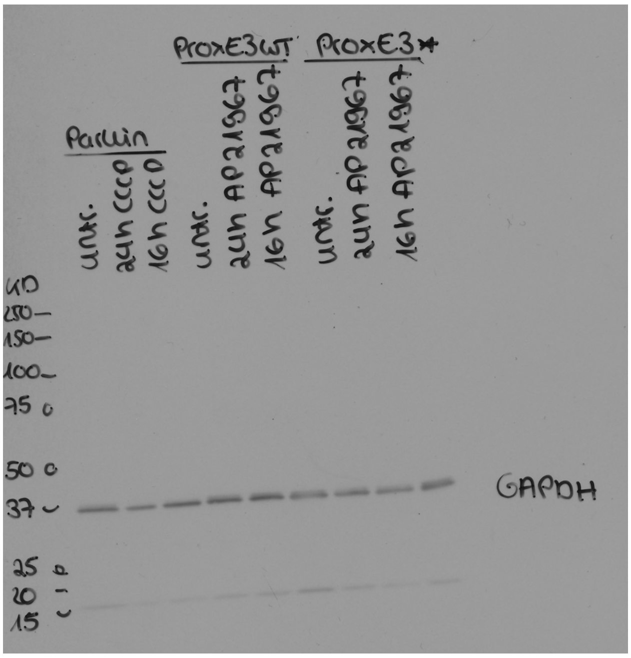

GAPDH detection (anti-GAPDH)

Figure 5 uncropped, unedited

B

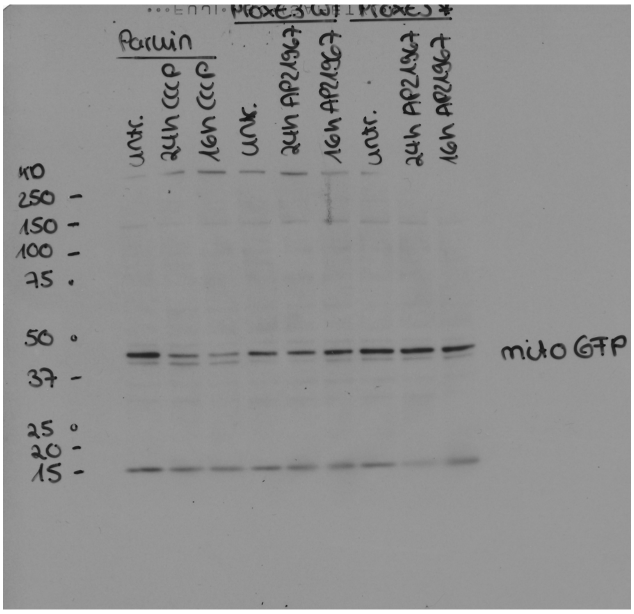

mitoGFP detection (anti-GFP)

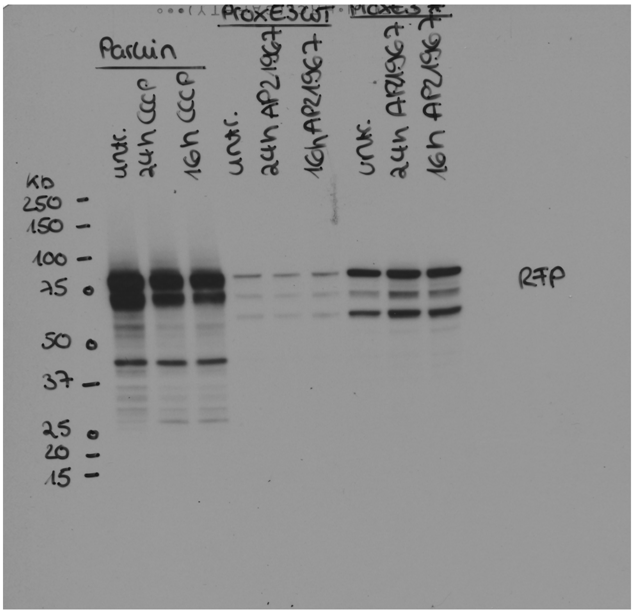

mCherry-detection (anti-RFP)

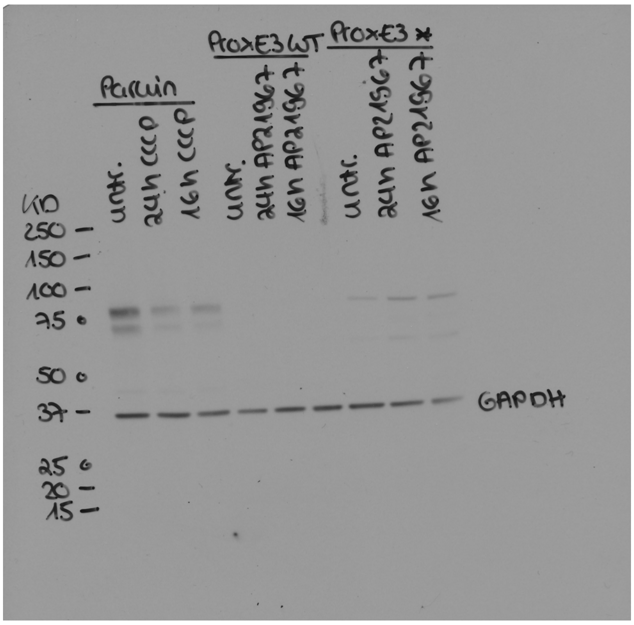

GAPDH detection (anti-GAPDH)
